# Genetic detection and analysis of porcine norovirus in pigs farmed in north Vietnam

**DOI:** 10.1016/j.heliyon.2024.e31946

**Published:** 2024-05-28

**Authors:** Hieu Van Dong, Giang Thi Huong Tran, Amonpun Rattanasrisomporn, Oumaporn Rungsuriyawiboon, Witsanu Rapichai, Jatuporn Rattanasrisomporn

**Affiliations:** a Faculty of Veterinary Medicine, Vietnam National University of Agriculture, Trau Quy Town, Gia Lam District, Hanoi, 131000, Viet Nam; b Interdisciplinary of Genetic Engineering and Bioinformatics, Graduate School, Kasetsart University, Bangkok, 10900, Thailand; c Department of Veterinary Technology, Faculty of Veterinary Technology, Kasetsart University, Bangkok, 10900, Thailand; d Department of Companion Animal Clinical Sciences, Faculty of Veterinary Medicine, Kasetsart University, Bangkok, 10900, Thailand

**Keywords:** Porcine norovirus, RNA-Dependent RNA polymerase gene, Pigs, PCR, Vietnam

## Abstract

Norovirus (NoV) causing gastroenteritis symptoms, which has been reported in several hosts, including humans, pigs, and rats. This study was conducted to identify porcine viral infection and to characterize NoV strains from pigs in some provinces in north Vietnam. Totally, 102 fecal samples from diarrheal pigs on farms in six cities and provinces in northern Vietnam during July 2022 to March 2023 were collected. Polymerase chain reaction was used to identify the viral genome. Positive samples were used for nucleotide sequencing of the partial RNA-dependent RNA polymerase gene sequence. Five (4.9 %) positive stool samples were detected from animals farmed in five different farms, with one positive animal identified in each farm. Genetic analysis indicated that nucleotide identity was in the range 97.77–99.62 % among the 5 NoVs in this study. Phylogenetic analysis pointed out that the five NoVs were Genotype II.19 viruses. Genetically, these strains were closely related to porcine NoV strains that were reported in China in 2009.

## 1 Introduction

Norovirus (NoV) has been identified as a main agent causing gastroenteritis symptoms in both humans and pigs [1]. In addition, NoVs have been reported as a leading pathogen of food-borne diseases [2]. Transmission routes of NoV can be fecal-oral, air-borne, water-borne, etc. [3]. Recovery of NoVs was successed in samples collected in both humans and animals, consisting of bovine, canine, pig, lion, and murine species [[4], [5], [6]]. NoV strains can infect all ages and cause moderate-to-severe diarrhea.

Analysis of the major capsid protein sequences, VP1, NoV belongs to the family *Caliciviridae* which forms into 10 genogroups, GI to GX. In addition, within the *Caliciviridae* family, 49 genotypes of NoV were classified [7]. NoVs are small, round, structured, non-enveloped viruses. The viral particle is approximately 27–38 nm in diameter. Their genomic composition encompasses a positive-sense and single-stranded genomic RNA. The length of viral genome ranges from 7.3 to 7.7 kb. The viral genetic material consists of three open reading frames (ORFs) [8], each responsible for encoding a polyprotein. ORF1 (∼5100 bp) is autocatalytically cleaved by viral protease resulting in the generation of 7 non-structural proteins (NS1–NS7). Among these, NS7 has been encoded for RNA-dependent RNA-polymerase (RdRp). Additionally, ORF2 (1600 bp) is responsible for the synthesis of viral pivotal capsid protein 1 (VP1). VP1 exhibits a structural organization comprising the shell (S) domain. The S singular the virus genome and the P domain contains the P2 domain. The P2 domain indicated as a highly genetic variability. The domain may contribute in inducing neutralization antibodies in the host [9]. In its capsid configuration, the S singular envelops the viral RNA and establishes a connection with the P1 and P2 domains through a hinge. ORF3 (∼720 bp) is responsible for encoding the minor capsid protein VP2, which contributes in viral assembly [10]. The genetics of NoVs are classified based on analysis of the full-length VP1 protein sequences and the ORF1 NS7 region nucleotide sequence [11].

In the field, porcine NoVs (PNoV) are formed within GII. GII NoVs were first reported among American pigs [12]. Subsequently, several countries have recorded the appearance of GII PNoVs in both sick and normal pigs [13]. The PNoV strains circulating in pigs were identified as GII.11, II.18, and II.19 genotypes in many countries in the world [[14], [15], [16], [17], [18], [19]]. Wang et al. reported that the Chinese PNoV strains belonged to three genotypes [12]. Among the three genotypes obtained, one genotype was antigenically and genetically related to the human norovirus [12]. However, the information on GII circulation and the characterization of NoV strains in pigs is still limited.

In Vietnam, NoV infection in humans was reported first in 1999 [20]. The NoV-positive rate in diarrheal children was 5.4 % (72/1339). Genetic analysis of the viral strains indicated that the obtained strains belonging to GII accounted for 73 %, with the remaining strains being GI.4, I.8, II.1, II.3, and II.7 [20]. In 2013, it was reported that 96.5 % (304/315) of the GII virus had been detected from May 2009 to December 2010 [21]. Until now, no data are available on the NoVs present in pigs. Hence, this study was conducted to investigate the presence of NoVs in pigs in north Vietnam.

## 2 Materials and methods

### 2.1 Ethics statement

Human participants were not involved in this study. Feces from pigs farmed in north Vietnam were sampled under the acceptance of the Vietnam National University of Agriculture (VNUA). The Committee on Animal Research and Ethics of the VNUA approved the sampling protocol (CARE-2022/08). In order collect samples, permissions of all the pig farm owners were given.

### 2.2 Samples

**Fig. 1:**
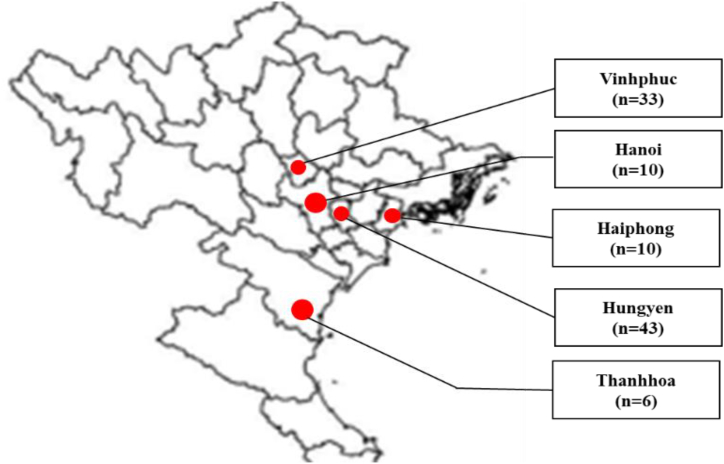
Geographical locations of sampling in north Vietnam. Locations of provinces and cities for sampling are indicated as red circles. Feces were sampled from pigs with diarrhea and dehydration farmed in Haiphong (n = 10), Hanoi (n = 10), Vinhphuc (n = 33) Thanhhoa (n = 6), and Hung Yen (n = 43).

Sows, fattening pigs, and piglets farmed in Haiphong (n = 10), Hanoi (n = 10), Vinhphuc (n = 33) Thanhhoa (n = 6), and Hung Yen (n = 43) were identified for sampling (Fig. 1). In total, three to six pigs with clinical sign of diarrhea and dehydration were sampled in each farm. Totally, 102 feces from pigs were sampled and immediately placed in separate sterile tubes, containing 1X phosphate-buffered saline (PBS) between July 2022 and March 2023. Samples were preserved in dry ice and sent to the Faculty of Veterinary Medicine of the Vietnam National University of Agriculture for further investigation. Next, the fecal samples were homogenized in PBS to prepare 10 % homonogenates and preserved at −80 °C.

### 2.3 Extraction of total RNA

Extraction of RNA was performed by using commercial GeneAll® Ribospin vRD II Kits (GeneAll Biotechnology; Gyeonggi-do, South Korea). The protocol was performed according to instructions of the manufacturer. A volume of 50 μl of distilled water was used to elute RNA. The tube containing total RNA was preserved at −80 °C.

### 2.4 Synthesis of cDNA

Performance of the cDNA synthesis was carried out by using a M-MLV reverse transcriptase (Invitrogen; Carlsbad, CA, USA). Reactions were conditioned thermally at 25 °C/10 min, 37 °C/60 min, 65 °C/10 min. The cDNA product was preserved at −30 °C.

### 2.5 Performance of PCR and nucleotide sequencing

PCR was performed to determine the PNoV, transmissible gastroenteritis virus (TGEV), porcine epidemic diarrhea virus (PEDV), porcine rotavirus (PoRoV), and porcine sapovirus genomes in the collected pig feces samples. The specific information of primers for each virus are indicated in Table 1. A partial RNA-dependent RNA polymerase (*RdRp*) gene region was amplified, previously described elsewhere [22]. PCR reagents were performed using GoTaq® Green Master Mix (Promega) and specifice primers (Table 1), followed by thermal conditions: of 94 °C/5 min, 35 cycles of 94 °C/30 s, 53 °C/60 s, and 72 °C/2.5 min, extension at 72 °C/10 min. The target PCR product of about 319 bp was electrophoresized on a 1.2 % agarose gel. Gel pictures were captured under ultraviolet radiation. Purification of the target PCR products was performed using a GeneClean® II Kit (MP Biomedicals; Santa Ana, CA, USA). The nucleotide sequencing was performed by the 1st BASE Company (Malaysia).

**Table 1:** Primers used for PCR in this study.

Target virus	Primer name	Nucleotide sequence (5' - 3′)	PCR product (bp)	Reference
Norovirus	P289	TGACAATGTAATCATCACCATA	319	[18]
	P290	GATTACTCCAAGTGGGACTCCAC		
Porcine rotavirus	Rot3	AAAGATGCTAGG GACAAAATTG	308	[23,24]
	Rot5	TTCAGATTGTGGAGCTATTCCA		
Porcine epidemic diarrhea virus	P1	TTCTGAGTCACGAACAGCCA	650	[24]
	P2	CATATGCAGCCTGCTCTG AA		
Transmissible gastroenteritis virus	T1F	GTGGTTTTGGTYRTAAATGC	859	[24]
	T1R	CACTAACCAACGTGGARCTA		
Porcine Sapovirus	PSaV-F	CTCATCAACCCTTTTGAAAC	757	[25]
	PSaV-R	AAAGCATGATGTTGTTAGGC		

### 2.6 Data analysis

Clustal W [26] in the BioEdit software [27] was applied to perform alignment and analysis of the nucleotide sequences in this study. The nucleotide homology among obtained sequences and other sequences from GenBank was performed using the Basic Local Alignment Search Tool (BLAST; https://blast.ncbi.nlm.nih.gov/) and GENETYX version 10 software (GENETYX Corp.; Tokyo, Japan) and nucleotide sequences of the Vietnamese PNoV strains in this study and other NoVs (43), Sapovirus (1), and Lagoviruse (2) (Table 2) from the GenBank database were used to construct a phylogenetic tree. A neighbor-joining method with the p-distance model was used to establish the tree in MEGA6 with 1000 replicates of bootstrapping [28]. Sequence data was available in GenBank with accession numbers OR835794–OR835798.

**Table 2:** Description of Norovirus, Sapovirus, and Lagovirus strains used to conduct genetic and phylogenetic characterization in this study.

Number	GenBank accession No.	Strain	Location	Source	Year	Virus
1	GU556171.1	SII-2-13	Taiwan	Pig	2008	Norovirus
2	GU556173.1	SII-2-16	Taiwan	Pig	2008	Norovirus
3	GU556169.1	SII-2-32	Taiwan	Pig	2008	Norovirus
4	GQ149616.1	sw42	China	Pig	2009	Norovirus
5	GQ149615.1	sw59	China	Pig	2009	Norovirus
6	HQ392821.1	Ch6	China	Pig	2009	Norovirus
7	AF194184.1	34	Netherlands	Pig	1998	Norovirus
8	JN644277.1	sw50	Netherlands	Pig	2009	Norovirus
9	JN644280.1	sw86	Netherlands	Pig	2010	Norovirus
10	MN605619.1	90186–1	Italy	Pig	2018	Norovirus
11	MN605620.1	90186–2	Italy	Pig	2018	Norovirus
12	FJ843083.1	Vet10-S08108	Denmark	Pig	2009	Norovirus
13	FJ843084.1	Vet14-S08257	Denmark	Pig	2009	Norovirus
14	FJ843087.1	Vet53-S08140	Denmark	Pig	2009	Norovirus
15	EU448332.1	F12-8	Canada	Pig	2005	Norovirus
16	EU448322.1	F12-8	Canada	Pig	2009	Norovirus
17	EU448323.1	F18-10	Canada	Pig	2009	Norovirus
18	EU448325.1	F16-8	Canada	Pig	2009	Norovirus
19	FJ498782.1	AB276	Canada	Pig	2009	Norovirus
20	FJ715807.1	31	Slovenia	Pig	2004	Norovirus
21	AB039775.1	Saitama U1	Japan	Human	2002	Norovirus
22	AB039776.1	Saitama U3	Japan	Human	2000	Norovirus
23	AB039777.1	Saitama U4	Japan	Human	2000	Norovirus
24	AB039781.1	Saitama U18	Japan	Human	2002	Norovirus
25	AB039780.1	Saitama U25	Japan	Human	2002	Norovirus
26	AB039782.1	Saitama U201	Japan	Human	2002	Norovirus
27	AY237415.2	Mc37	Japan	Human	2004	Norovirus
29	AB074892.1	Sw43	Japan	Pig	1997	Norovirus
30	AB126320.1	swine43	Japan	Pig	2009	Norovirus
31	AF145896.1	Camberwell/101922	Australia	Human	1994	Norovirus
32	X86557.1	Lordsdale	UK	Human	2002	Norovirus
33	AY772730.1	Neustrelitz260	Germany	Human	2000	Norovirus
34	U07611.2	Hawaii virus	USA	Human	1971	Norovirus
35	AY038599.2	VA97207	USA	Human	1997	Norovirus
36	AY823304.1	OH-QW101	USA	Pig	2003	Norovirus
37	AY823305.2	OH-QW125	USA	Pig	2003	Norovirus
38	AY823306.1	OH-QW170	USA	Pig	2003	Norovirus
39	AY823307.1	OH-QW218	USA	Pig	2003	Norovirus
40	AF097917.5	Newbury2	UK	Bovine	1976	Norovirus
41	AF182760.1	Cowden virus	USA	Pig	1999	Sapovirus
42	M67473.1	RHDV-FRG	Germany		1991	Lagovirus
43	Z69620.1	EBHSV-GD	France		1996	Lagovirus

### 2.7 Statistical analysis

Comparison of rates of the PNoV genome detection according to sampling area, age, and scale of pig farms was performed using Fisher's exact test. A p-value less than 0.05 was used to test for statistically significant differences.

## 3 Results

### 3.1 Detection of PNoV genome in field samples

The fecal samples tested were obtained from pigs indicating clinical signs of diarrhea and dehydration. Of 102 pigs examined, the viral genome was detected in five (4.9 %) samples by PCR analysis. The porcine rotavirus, PEDV, TGEV, and porcine sapovirus genome were not detected in these five samples. The PNoV genome-positive rates were 10 %, 10 %, and 6.98 % (p > 0.05) in Hanoi, Haiphong, and Hungyen, respectively, while no positive samples were detected in the remaining two provinces (Thanhhoa and Vinhphuc). Of the 20 pig farms tested, 5 (20 %) were positive for at least one sample to the detection of norovirus. The genome-positive rates were 50 % and 50 % in Hanoi and Haiphong, respectively, and then for Hungyen (37.5 %) (p > 0.05) (Table 3).

**Table 3:** Identification of porcine norovirus genome in fecal samples from pigs in North Vietnam using PCR.

Province/city	No. of collected samples	No. of gene-positive samples (%)	No. of farms	No. of gene-positive farms (%)
Hanoi	10	1 (10.00)	2	1 (50.00)
Haiphong	10	1 (10.00)	2	1 (50.00)
Hungyen	43	3 (6.98)	8	3 (37.50)
Thanhhoa	6	0 (0.00)	1	0 (0.00)
Vinhphuc	33	0 (0.00)	7	0 (0.00)
Total	102	5 (4.90)	20	5 (25.00)

Based on age, 4 (12.12 %) samples were positive for the PNoV genome in finishing pigs, which was significantly higher (p = 0.02) than for a postweaning pig at ten weeks of age (3.03 %). There were no positive samples in the piglets (<21 days of age) or sows. Three levels of farm scale were classified: level 1 (<100 pigs), level 2 (100–300 pigs), and level 3 (>300 pigs). Three (15 %) samples were positive for the viral genome at farm level 2, which was significantly higher (p = 0.02) than for farm level 1 (3.85 %). No positive samples were detected in pig farms at level 3 (Table 4).

**Table 4:** Identification of PNoV genome in fecal samples of pigs according to pig age and farm scale.

Parameter	Age (Week)	Number of tested samples	Number of gene-positive samples(%)
Pig age class			
Nursing	<3	6	0 (0.00)
Postweaning	3–12	33	1 (3.03)^a^
Finishers	18–24	33	4 (12.12)^b^
Sows	>50	30	0 (0.00)
Farm scale			
< 100		52	2 (3.85)^x^
100–300		20	3 (15.00)^y^
> 300		30	0 (0.00)

Different lowercase superscripts ^a, b, x, and y^ indicate significant differences between groups (p < 0.05).

### 3.2 Genetic and phylogenetic analysis of PNoV strains

The five Vietnamese strains were named as Vietnam/PNoV/VNUA-06, -22, −35, −40, and −56. Among these Vietnamese viral strains, nucleotide identity was high, ranging from 97.77 % (Vietnam/PNoV/VNUA-35 vs. Vietnam/PNoV/VNUA-40 and VNUA-56) to 99.62 % (Vietnam/PNoV/VNUA-40 vs. Vietnam/PNoV/VNUA-56) (Table 5). Comparisons of the five Vietnamese PNoVs from the current study with other viruses downloaded from the GenBank database indicated that these viruses shared the highest nucleotide identities of 98.88 % (Vietnam/PNoV/VNUA-40 vs. Pig/China/GII/Ch6/2009 (HQ392821.1) and 99.25 % (Vietnam/PNoV/VNUA-06, Vietnam/PNoV/VNUA-35 vs. Pig/China/GII.19/Sw42-09-Ch (GQ149616.1); Vietnam/PNoV/VNUA-22 vs Pig/China/GII/Ch6/2009 (HQ392821.1); Vietnam/PNoV/VNUA-56 vs. Pig/China/GII/Sw59-09-Ch/2009 (GQ149615.1)) (Table 6).

**Table 5:** Comparisons of nucleotide identity of partial *RdRp* gene (270 bp) among sequences of five Vietnamese PNoV strains.

Strain name	Nucleotide identity (%)				
	Vietnam/PNoV/VNUA-06	Vietnam/PNoV/VNUA-22	Vietnam/PNoV/VNUA-35	Vietnam/PNoV/VNUA-40	Vietnam/PNoV/VNUA-56
Vietnam/PNoV/VNUA-06	100				
Vietnam/PNoV/VNUA-22	98.51	100			
Vietnam/PNoV/VNUA-35	99.25	99.25	100		
Vietnam/PNoV/VNUA-40	98.14	98.51	97.77	100	
Vietnam/PNoV/VNUA-56	98.51	98.51	97.77	99.62	100

**Table 6:** Comparisons of nucleotide identity of partial *RdRp* gene (270 bp) of sequences of five Vietnamese PNoV strains with downloaded sequences from GenBank database.

Number	Strain name	Virus with highest nucleotide identity				
		Strain	Country	Accession number	Year	Identity (%)
1	Vietnam/PNoV/VNUA-06	Sw42	China	GQ149616.1	2009	99.25
2	Vietnam/PNoV/VNUA-22	Ch6	China	HQ392821.1	2009	99.25
3	Vietnam/PNoV/VNUA-35	Sw42	China	GQ149616.1	2009	99.25
4	Vietnam/PNoV/VNUA-40	Ch6	China	HQ392821.1	2009	98.88
5	Vietnam/PNoV/VNUA-56	Sw59	China	GQ149615.1	2009	99.25

**Fig. 2:**
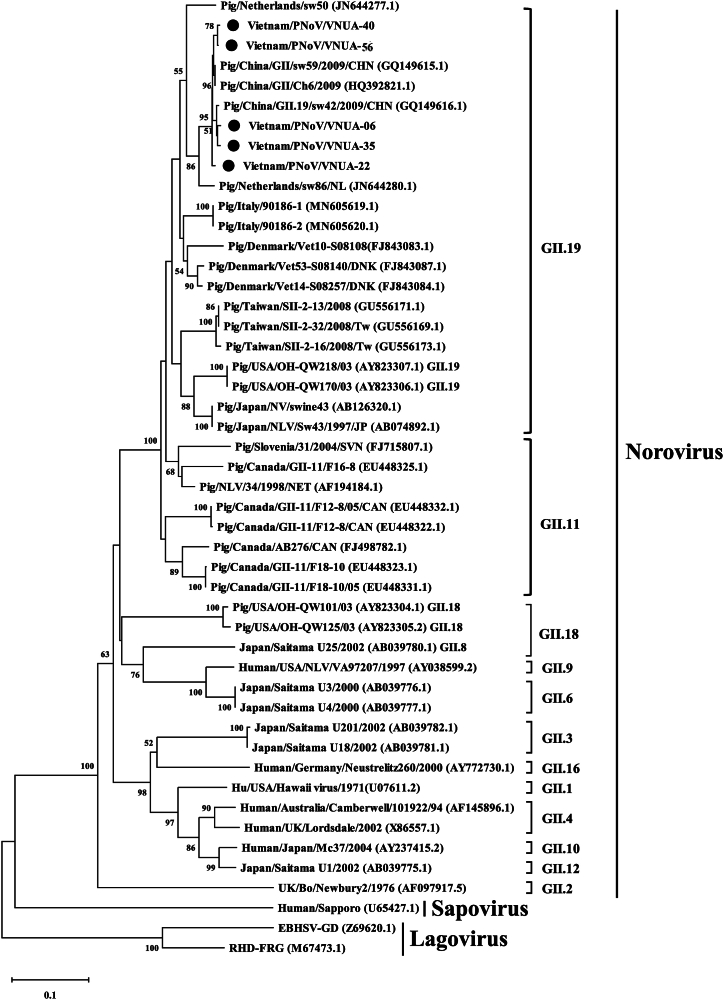
Neighbor-joining phylogenetic tree of *RdRp* gene (270 bp) sequences of current Vietnamese porcine norovirus strains and sequences from GenBank database, where MEGA 6 software [28] was used with 1000 bootstrap replicates and the five Vietnamese viral strains are indicated with filled black circles.

In order to construct a phylogenetic tree, the partial *RdRp* gene sequence (270 bp) of the five viral strains obtained in the current study and 34 other viruses were used as reported previously [29,30]. The phylogenetic tree showed that the Vietnamese strains were PNoVs that could be divided into 2 sub-clusters. These five Vietnamese PNoVs obtained were clustered with Chinese PNoV strains Pig/China/GII.19/sw42/2009 (GQ149616.1), Pig/China/GII/sw59/2009 (GQ149615.1), and Pig/China/GII/Ch6/2009 (HQ392821.1) (Fig. 2), which belonged to Genotype II.19.

## 4 Discussion

PNoV circulates among several hosts, including humans and animals (such as pigs, mice, dogs, and sheep) [31]. PNoV infection was reported in several countries in the world, including Vietnam [21]. There are currently only limited Vietnamese PNoV sequences in GenBank. To our knowledge, this study was the first report on PNoV infection in north Vietnam in pigs and on the molecular characteristics of the viral strains in the country.

In this study, the PNoV genome was detected in 5 (4.9 %) out of 102 fecal samples from pigs with signs of diarrhea and dehydration. This rate was somewhat low, being similar to that in several Asian countries, Korea (0.5 %) [32], Taiwan (7.2 %) [33], and Japan (10 %) [34], and in European countries, Slovenia (1.2 %) [35] and Belgium (4.6 %), but lower than in the USA (20 %) [36] and Canada (25 %) [5]. These differences in the rates of PNoV-positive samples could be due to variations in location, time, sensitivity of the identification method, and the number of tested samples. Additionally, of the 20 pig farms tested, the viral genome was detected in 5 (25 %) in north Vietnam, suggesting that PNoV strains may have been affecting the porcine production industry in the country. The present result found that only one fecal sample from each farm was detected to be positive. As mentioned previously, several ways of norovirus transmission were recorded, including fecal-oral or via air-borne routes, etc. [3]. This was due to the fact that sample size, sampling scheme may affect the result number of positive samples in each pig farm. Further study would be perform to expand numbers of samples in each farm, numbers of farms to clarify this point. Previous studies reported that PNoV could be detected in both sick and healthy pigs [33,37]. In experimental study, gnotobiotic piglets infected with PNoV developed mild diarrhea at five day-post inoculation [38]. Affected piglets continued shedding virus during viral infection [29,38]. PNoV infection was detected in asymptomatic swine [33,39]. Until now, no direct evident showed that PNoV plays critical roles on raising of gastroenteritis in natural cases of infection in pigs. Clinical signs of affected pigs were non-specific and it was hard to diagnose. The current study has reported PNoV in diarrheal pigs for the first time in Vietnam. Although PNoV-positive samples were tested and negative for the porcine rotavirus, PEDV, TGEV, and PSaV genomes, other pathogens including viruses, bacteria, etc. would be involved to be examined to clarify the role of PNoV strains. Additional studies should be conducted to isolate viral strains and accessed pathogenicity of the current Vietnamese PNoV strains in this study.

Regarding age of infection, Wang et al. (2006) reported that PNoV infection was detected in fecal samples in 43 (40.95 %) out of 105 samples collected from finishers in the U.S., whereas they could not detect PNoV-positive samples from nursing and postweaning pigs during 2003–2005 [36]. In this study, the PNoV genome was identified in a postweaning pig at ten weeks of age (3.03 %), Finishers (12.12 %), whereas no positive samples were found in piglets (<21 days-old) and sows. Taken together, young piglets could not be susceptible to natural PNoV infection. However, Chao et al. noted that suckling (9.2 %), weaning (5.4 %) and finishing (7 %) pigs farmed in central and southern Taiwan in 2011 were positive for the PNoV genome [33]. Further studies should be conducted to expand sampling numbers to better estimate the age-related percentages of PNoVs among Vietnamese pigs. In addition, current results noted that PNoV-positive rates were different between farm scale, suggesting that management of animals may affect rates of PNoV infection in pig farms. No direct evidence indicated the presence of NoV to be associated with the management of animals in the investigated farms more than to their age in this study.

For genetic characterization, previous studies classified NoVs using the partial or complete ORF1 NS7 region that codes for the partial RdRp protein [30,[23], [40], [41]]. Among genotypes, GII genotype viruses were detected as circulating widely in Asian countries, with strains detected broadly across pig farms [12,24]. Genetic and phylogenetic analyses pointed out that the five Vietnamese PNoVs and other Chinese viruses (Pig/China/GII.19/sw42/2009, Pig/China/GII/sw59/2009, and Pig/China/GII/Ch6/2009) were clustered in a similar genotype and belonged to porcine norovirus genotype II.19 (Fig. 2). The current results obtained suggested that PNoV genotype II continues to be widely spread among Asian countries.

Norovirus spreads and circulates in both humans and animals, which raises a potential risk of a zoonotic disease due to direct close contact. Genotype II strains have been widely identified in humans and several animal species [12,24], with genotypes II.11, II.18, and II.19 being specific for pigs. In the current study, all five PNoV strains were genotype II.19 viruses. We found no relationships between the obtained Vietnamese PNoV strains to human NoVs. Further study should continue to determine any associations among the NoVs circulating in pigs and humans in Vietnam.

PNoV infection was detected not only in diseased pigs but also healthy pigs [33,37]. In this study, only pigs with diarrheal symptoms were studied, but not healthy pigs. These current data may not indicated an overlook of PNoV among pigs in Vietnam. More samples from healthy pigs would be collected and conducted in the future. In term of genetic and phylogenetic characterization, previous studies used the partial and full-length RdRp sequences of PNoV strains characterize the viral strains [29,30,33]. Meanwhile, the full-length VP1 and complete genome were also used to conduct genetic characteristics of the PNoVs [[25], [42], [43]]. In this study, only a short RdRp gene (270 bp) of Vietnamese PNoVs were characterized. Further studies should be developed to get the complete VP1 gene and genome of the Vietnamese PNoV strains to perform genetic analysis deeply.

## 5 Conclusions

To our knowledge, this was the first study on PNoV infection in north Vietnam. The norovirus genome-positive rate was low (4.9 %), while 25 % of the sampled farms produced positive results in the present study. The five sequences obtained belonged to porcine norovirus GII.19, which has spread widely in pigs. The Vietnamese PNoVs were closely related to the Chinese PNoV strains. This study identified no evidence of any human norovirus among the Vietnamese pigs farmed in north Vietnam.

## Funding

The research was supported by the Vietnam National University of Agriculture, Vietnam under grant number T2023-09-36, and the Faculty of Veterinary Medicine, 10.13039/501100004539Kasetsart University, Thailand and by the 10.13039/501100004704National Research Council of Thailand (10.13039/501100004704NRCT) through an 10.13039/501100004704NRCT Senior Scholarship to R. Thanawongnuwech (2022 #N42A650553).

## CRediT authorship contribution statement

**Hieu Van Dong:** Writing – original draft, Software, Methodology, Investigation, Funding acquisition, Formal analysis, Data curation, Conceptualization. **Giang Thi Huong Tran:** Software, Methodology, Investigation. **Amonpun Rattanasrisomporn:** Writing – review & editing, Resources. **Oumaporn Rungsuriyawiboon:** Writing – review & editing. **Witsanu Rapichai:** Writing – review & editing, Writing – original draft. **Jatuporn Rattanasrisomporn:** Writing – review & editing, Writing – original draft, Visualization, Supervision, Resources, Project administration, Investigation, Formal analysis, Data curation, Conceptualization.

## Declaration of competing interest

The authors declare that there was no conflict of interest.
